# Clinical validity of Midway PGT-A: a comparative study with traditional PGT-A and analysis of fertilization strategies

**DOI:** 10.1530/RAF-25-0128

**Published:** 2026-08-14

**Authors:** Jiujie Dou, Zhan Hu, Boya Zhao, Senlin Shi

**Affiliations:** ^1^ Center for Reproductive Medicine, The First Affiliated Hospital of Zhengzhou University, Zhengzhou, China; ^2^ Henan Key Laboratory of Reproduction and Genetics, The First Affiliated Hospital of Zhengzhou University, Zhengzhou, China; ^3^ Henan Provincial Obstetrical and Gynecological Diseases (Reproductive Medicine) Clinical Research Center, The First Affiliated Hospital of Zhengzhou University, Zhengzhou, China; ^4^ Henan Engineering Laboratory of Preimplantational Genetic Diagnosis and Screening, The First Affiliated Hospital of Zhengzhou University, Zhengzhou, China

**Keywords:** Midway PGT-A, traditional PGT-A, euploidy, double vitrification, clinical outcomes

## Abstract

**Graphical Abstract:**

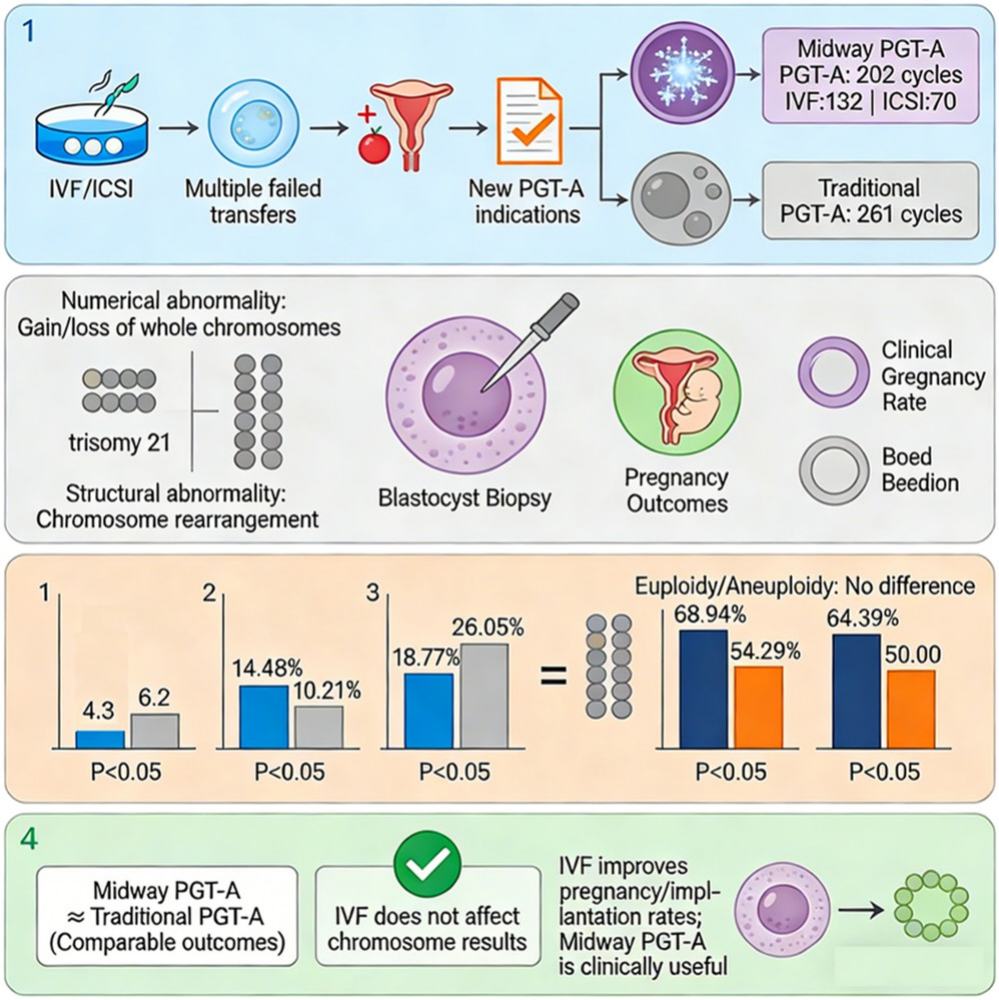

**Abstract:**

Infertile patients initially undergoing *in vitro* fertilization/intracytoplasmic sperm injection (IVF/ICSI) with fresh embryo transfer often lack baseline indications for preimplantation genetic testing for aneuploidy (PGT-A); however, after multiple failed transfers, they may become eligible for PGT-A, which can be performed on residual vitrified–thawed embryos (a strategy termed ‘Midway PGT-A’), whose clinical value remains unconfirmed. This study aimed to assess the clinical utility of Midway PGT-A and whether fertilization methods (IVF vs ICSI) affect its chromosomal testing results and clinical outcomes. A 2020–2023 retrospective cohort study analyzed 463 PGT-A cycles, comprising 202 Midway PGT-A cycles (132 IVF and 70 ICSI subcycles) and 261 traditional PGT-A cycles, with comparisons made regarding blastocyst aneuploidy profiles and clinical pregnancy outcomes. The results showed that compared with traditional PGT-A, Midway PGT-A was associated with fewer biopsied blastocysts (*P* < 0.05), a higher rate of numerical chromosomal abnormalities (*P* < 0.05), and a lower rate of structural chromosomal abnormalities (*P* < 0.05). In the Midway PGT-A group, IVF and ICSI subcycles had similar euploidy and aneuploidy rates, but the IVF subcycle exhibited a significantly higher clinical pregnancy rate (*P* < 0.05) and implantation rate (*P* < 0.05). Multivariable logistic regression confirmed that ICSI was independently associated with a lower clinical pregnancy rate compared with IVF. In summary, Midway PGT-A delivers equivalent outcomes compared with traditional PGT-A. IVF does not alter the chromosomal detection results of Midway PGT-A and may further optimize clinical reproductive performance, validating the clinical value of Midway PGT-A for patients with post-transfer PGT-A eligibility.

**Lay summary:**

Many people undergoing fertility treatments do not need embryo genetic testing initially. However, after repeated failed pregnancies, miscarriages, or having a baby with birth defects, they may need this test later. We tested the genetic health of their leftover frozen embryos – a strategy we call ‘Midway testing’. Our study of 463 cycles found that Midway testing worked as well as standard testing done earlier. The number of embryos tested was lower for Midway testing, but it found more whole-chromosome issues and fewer chromosome structure issues. Fertilization methods did not change test results, but one method led to higher pregnancy and implantation rates. This testing offers a valuable option for people who need genetic embryo checks after failed fertility cycles, helping them use existing frozen embryos effectively.

## Introduction

Infertility affects approximately 15% of couples of reproductive age worldwide, and *in vitro* fertilization (IVF) or intracytoplasmic sperm injection (ICSI) has become the mainstay of treatment for these populations (Mascarenhas *et al.* 2012). However, the success rate of IVF/ICSI remains suboptimal, as chromosome aneuploidy – the main cause of embryo arrest, implantation failure, recurrent miscarriage (RM), and birth defects (Bodri *et al.* 2015) – poses a major clinical challenge. Preimplantation genetic testing for aneuploidy (PGT-A) is a key assisted reproductive technology that screens for chromosomal aneuploidies in embryos before transfer, thereby improving the efficiency of embryo selection and reducing the risk of adverse pregnancy outcomes. It is critical to clarify core definitions in this context: traditional PGT-A refers to genetic testing performed on embryos immediately after *in vitro* culture, based on preestablished clinical indications at the initiation of the IVF/ICSI cycle; Midway PGT-A, as defined in this study, specifically refers to the scenario where patients do not meet PGT-A indications at baseline when undergoing initial IVF/ICSI with fresh embryo transfer, but develop new PGT-A indications (e.g. after two or more repeated implantation failures (RIFs), two or more RMs, or the birth of a malformed fetus with chromosomal abnormalities) following failed transfer attempts. For these patients, the remaining frozen embryos from the initial cycle are thawed for PGT-A, which means euploid blastocysts in Midway PGT-A undergo two rounds of vitrification and thawing/warming before transfer – a key distinction from traditional PGT-A.

The clinical value of traditional PGT-A has been well validated by accumulating evidence. A study found that PGT-A did not improve the live birth rate per patient, but it did have the advantage of reducing the number of embryo transfers required to achieve a similar number of live births compared with those not undergoing PGT-A (Sato *et al.* 2019). A meta-analysis further confirmed that PGT-A enhances LBR per transfer and per patient in unexplained RPL (Mumusoglu *et al.* 2025). These findings collectively support that PGT-A plays a crucial role in optimizing reproductive outcomes by excluding aneuploid embryos. Notably, traditional PGT-A often uses ICSI as the insemination method to avoid contamination of embryo biopsy samples by paternal (sperm) and maternal (granulosa cell) genetic material. IVF and ICSI differ in fertilization mechanisms, and previous studies have suggested that in couples with infertility with a non-severe male factor, ICSI did not improve live birth rate compared with conventional IVF (Wang *et al.* 2024). However, conventional IVF is also a common fertilization method in Midway PGT-A patients, and whether IVF affects the accuracy of chromosomal testing results and subsequent clinical outcomes remains unclear. Reinitiating IVF/ICSI imposes physical, emotional, and economic burdens, making residual frozen embryos a valuable resource. While vitrification advances (Rienzi *et al.* 2017) have boosted frozen embryo transfer (FET) success, critical gaps persist: i) does Midway PGT-A (with double vitrification/thawing) achieve outcomes comparable to traditional PGT-A? ii) Do fertilization methods (IVF vs ICSI) influence its testing accuracy or clinical effectiveness? Current research on PGT-A focuses on traditional strategies (Hu *et al.* 2024, Mumusoglu *et al.* 2025), with limited evidence on Midway PGT-A; existing studies lack systematic comparison with traditional PGT-A or IVF/ICSI subgroup analyses. Validating Midway PGT-A would provide a more personalized treatment option for patients who develop PGT-A indications post-transfer, avoiding unnecessary cycle repetition, improving the utilization rate of existing embryos, and ultimately optimizing the management of infertile patients with failed transfer histories. This study aimed to address the aforementioned research gaps with the following research questions: what is the clinical application value of Midway PGT-A compared with traditional PGT-A? Specifically, do two rounds of cryopreservation/thawing affect the outcomes of Midway PGT-A? And do different fertilization methods (IVF vs ICSI) influence its chromosomal testing results and clinical outcomes? The findings will provide evidence-based support for the clinical application of Midway PGT-A and optimize personalized treatment strategies for infertile patients with failed transfer histories.

## Materials and methods

### Study population

**Figure 1:**
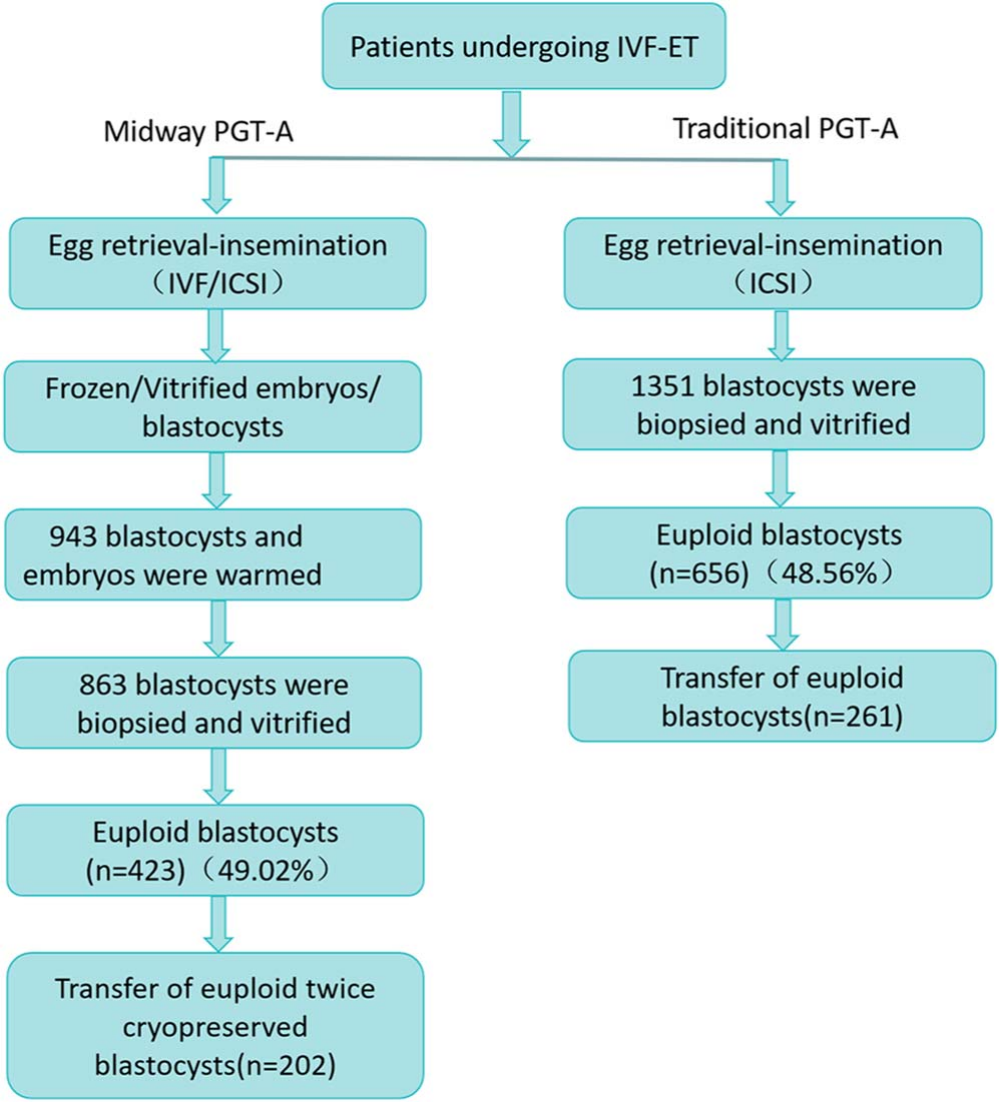
Flow chart of the study design. IVF–ET: *in vitro* fertilization and embryo transfer. IVF: *in vitro* fertilization. ICSI: intracytoplasmic sperm injection. PGT-A: preimplantation genetic testing for aneuploidy. In the Midway PGT-A group, a total of 943 embryos/blastocysts were thawed. Eighty embryos were excluded due to post-thaw death or failure to develop into blastocysts, and 863 eligible blastocysts were subjected to PGT-A biopsy.

This retrospective cohort study was conducted using data from the Clinical Reproductive Medicine Management System Medical Record Cohort Database at the Reproductive Center of the First Affiliated Hospital of Zhengzhou University. We compared clinical outcomes between the Midway PGT-A group and the traditional PGT-A group. A total of 463 PGT-A cycles performed between January 2020 and January 2023 were enrolled and divided into two groups (Fig. 1): the first was the Midway PGT-A group: 202 cycles with 863 blastocysts, consisting of patients who had residual cryopreserved embryos/blastocysts from initial IVF/ICSI cycles and subsequently underwent PGT-A after developing new indications (e.g. ≥2 repeated implantation failures (RIFs), ≥2 RMs, or birth of a malformed fetus with chromosomal abnormalities). The fertilization modality (IVF or ICSI) applied in the original cycles was determined strictly according to male semen quality: conventional IVF was performed for patients with normal semen parameters, whereas ICSI was reserved exclusively for cases with severe male factor infertility, consistent with standard clinical guidelines. The fertilization decision in this cohort was not influenced by the personal preferences of clinicians or embryologists.

The second was the traditional PGT-A group: 261 cycles with 1351 blastocysts, consisting of patients who underwent trophectoderm biopsy and PGT-A immediately after embryo culture based on baseline clinical indications. The traditional PGT-A group also met the standard clinical indications for PGT-A, including ≥2 repeated implantation failures (RIFs), ≥2 RMs, or a prior pregnancy complicated by chromosomally abnormal fetal malformation.

#### Exclusion criteria

The exclusion criteria were as follows: i) female infertility-related factors, such as uterine malformations, endometriosis, and ovarian dysfunction. ii) Severe male factors, including high sperm DNA fragmentation index (DFI). (In this study, sperm DNA fragmentation (SDF) was systematically measured in all male partners using the sperm chromatin structure assay (SCSA). SDF > 30% was defined as the exclusion threshold. This cutoff was adopted based on clinical evidence (Fu *et al.* 2023); cases with SDF > 30% were excluded to minimize the confounding effect of high SDF on embryo ploidy and clinical outcomes.) iii) Abnormal karyotypes in either partner.

### *In vitro* fertilization and embryo culture

#### Midway PGT-A group

Transvaginal ultrasound-guided oocyte retrieval was conducted. Fertilization was performed 39–40 h after human chorionic gonadotropin (HCG) administration. Two fertilization strategies were used based on male semen parameters. The IVF protocol contained the following steps: oocytes were co-incubated with sperm, cumulus cells were removed using a 150–160 μm denuding needle (Sunlight, USA) at 4–6 h post-fertilization, and fertilization was confirmed by visualization of the second polar body extrusion. The ICSI protocol contained the following steps: cumulus cells were denuded with hyaluronidase (Sage, USA) one hour after oocyte retrieval, followed by the ICSI procedure one hour later. All embryos were cultured in sequential culture media (Vitrolife, Sweden; Cook, Australia; Sage, USA). Embryo morphology and quality were evaluated by experienced embryologists on day 3 and on day 5 or day 6 after fertilization. Surplus embryos after embryo transfer were preserved via vitrification.

#### Traditional PGT-A group

Compared with the Midway PGT-A group, the traditional PGT-A group underwent ICSI treatment irrespective of their sperm quality. Embryos were cultured to day 5 or day 6 for blastocyst biopsy before vitrification. All other embryo culture conditions and procedures were identical between the two groups.

### Trophectoderm biopsy

#### Midway PGT-A group

Biopsied blastocysts were derived from two sources: i) day 3 vitrified embryos that were thawed and cultured to day 5 or day 6 and ii) vitrified blastocysts cryopreserved on day 5 or day 6. Thawing was performed following the manufacturer’s instructions. Biopsy procedures were standardized by the following blastocyst stages:

i) Day 5 or day 6 blastocysts (stages 5–6): 3–5 trophectoderm cells were aspirated from the region distant from the inner cell mass (ICM).

ii) Day 5 or day 6 blastocysts (stages 3–4): a small hole was drilled in the zona pellucida (distant from the ICM, with no visible sperm or granulosa cell contamination) using the OCTAX laser system 1–2 h before biopsy; 3–5 expanded/hatched trophectoderm cells were then extracted.

Post-biopsy, blastocysts were immediately transferred to a four-well plate (Oosafe, Denmark) and incubated at 37°C with 6% CO_2_ for subsequent vitrification. Trophectoderm cells were collected into PCR tubes for genetic analysis.

#### Traditional PGT-A group

On day 4 post-fertilization, a laser-drilled hole was made in the zona pellucida (distant from the ICM) using the OCTAX laser system. On day 5 or day 6, 3–5 expanded/hatched trophectoderm cells were aspirated. Post-biopsy, processing of blastocysts and trophoblast cells was identical to the Midway PGT-A group.

### Blastocyst vitrification after biopsy

Vitrification was performed using the Kitazato® Vitrification Kit (Kitazato, Japan) and VitaVitro® Vitrification Kit (VitaVitro, China). Equilibration solution (ES) and vitrification solution (VS) were pre-equilibrated at room temperature for 30 min. Blastocysts were processed as follows:

Blastocysts were incubated in ES for 8–10 min and then in VS for 45–60 s.

Next, they were loaded onto cryotops with minimal residual liquid, immediately plunged into liquid nitrogen for rapid cooling, and then stored in liquid nitrogen tanks with labeled identifiers.

### NGS testing and data analysis

#### Next-Generation Sequencing (NGS) and data analysis

Trophectoderm cell samples were analyzed using the HumanCytoSNP-12 DNA Array (Illumina, USA) following the manufacturer’s protocol (XK028, Yikon Genomics, China). Data were processed using Illumina Genome Studio and KaryoStudio software. Embryos were classified into four mutually exclusive categories according to the classification standard reported in the relevant literature (Niu *et al.* 2020): euploid embryos (46, XN), embryos with numerical chromosomal abnormalities, embryos with structural chromosomal abnormalities, and embryos with complex multiple chromosomal abnormalities. Only euploid embryos were subjected to embryo transfer.

### Blastocyst warming and transfer

Euploid blastocysts were selected for warming using the Kitazato® Vitrification Thaw Kit (Kitazato, Japan) and the VitaVitro® Vitrification Thaw Kit (VitaVitro, China). Thawing solution (TS) was pre-equilibrated at 37°C for ≥2 h; dilution solution (DS), washing solution 1 (WS1), and washing solution 2 (WS2) were prepared at room temperature .

Blastocysts were removed from cryotops and incubated in TS for 1 min, DS for 3 min, WS1 for 5 min, and WS2 for 3 min.

Warmed blastocysts were cultured in blastocyst culture medium at 37°C with 6% CO_2_ before single blastocyst transfer.

### Endometrial preparation for FET

A single thawed euploid embryo was transferred for each transfer cycle. Only euploid embryos were used for FET in this cohort. Endometrial preparation for FET was performed via either natural cycle or hormone replacement therapy (HRT) cycle. The regimen selection was based on patients’ menstrual regularity and ovulatory function. Patients with regular spontaneous ovulation underwent natural cycles, with follicle development and ovulation monitored by transvaginal ultrasound. For patients with irregular menstruation or ovulatory dysfunction, HRT cycles were adopted: oral estradiol was given to promote endometrial growth, and progesterone was supplemented once the endometrial thickness reached ≥8 mm prior to embryo transfer.

### Outcome measures and clinical outcomes

#### Primary outcome

The primary outcome was the embryo euploidy/aneuploidy rate, calculated as the number of euploid/aneuploid blastocysts divided by the total number of biopsied blastocysts.

#### Secondary outcomes

The secondary outcomes were clinical pregnancy rate, implantation rate, miscarriage rate, and live birth rate. They were defined as follows: clinical pregnancy rate = (number of cycles with ultrasound-confirmed gestational sac at 35 days post-transfer)/(total number of transfer cycles); implantation rate = (number of gestational sacs)/(number of transferred embryos); miscarriage rate = (number of miscarriages before 12 weeks of gestation)/(number of clinical pregnancy cycles); and live birth rate = (number of live births)/(number of transferred embryos).

### Statistical analysis

Data were analyzed using SPSS 21.0 software. Continuous variables are presented as mean ± standard deviation, and categorical variables are presented as percentages. Intergroup comparisons were made using Student’s *t*-test (parametric data), the Mann–Whitney U test (non-parametric data), or the chi-square test (categorical data). Multivariable binary logistic regression was performed to identify independent predictors of clinical pregnancy, with clinical pregnancy as the binary dependent variable. All clinically relevant covariates with potential impacts on reproductive outcomes were prespecified and simultaneously entered into the full model based on prior reproductive medicine evidence, including fertilization modality (IVF vs ICSI), maternal age, paternal age, BMI, duration of infertility, AMH, and endometrial thickness; automated stepwise variable selection was not used to eliminate selection bias. Statistical significance was set at *P* < 0.05.

## Results

### Baseline characteristics of all subjects

A total of 463 couples who underwent PGT-A were eligible for the final analysis, including 202 couples in the Midway PGT-A group and 261 couples in the traditional PGT-A group (Table 1). There were no statistically significant differences in baseline characteristics between the two groups, including female age, female body mass index (BMI), male age, duration of infertility, PGT-A indications, basic endocrine parameters, anti-Müllerian hormone (AMH) level, and endometrial thickness at transfer (*P* > 0.05).

**Table 1:** Basic characteristics of Midway PGT-A group and traditional PGT-A group. Data are presented as mean ± SD or as *n* (%). Intergroup comparisons were made using Student’s *t*-test (parametric continuous variables), the Mann–Whitney U test (non-parametric continuous variables), or the chi-square test (categorical variables). Statistical significance was defined as *P* < 0.05.

Characteristics	Midway PGT-A group	Traditional PGT-A group	Statistics	
			*χ*^2^/*t*	*P*
Cycles, *n*	202	261		
Female age, years	33.19 ± 4.42	33.40 ± 4.21	1.35	0.584
Male age, years	34.03 ± 4.76	34.54 ± 5.63	−1.16	0.25
Female BMI, kg/m^2^	23.42 ± 3.48	23.10 ± 2.96	1.07	0.14
Infertility, years	3.50 ± 2.59	3.34 ± 2.47	0.76	0.22
PGT-A indications, *n* (%)				
Recurrent implantation failure	39/202 (19.31%)	45/261 (17.24%)	0.33	0.57
Recurrent miscarriage	153/202 (75.74%)	209/261 (80.08%)	1.25	0.26
Fetal malformation	10/202 (4.95%)	7/261 (2.68%)	1.66	0.20
Basic endocrine parameters				
Basic FSH, mIU/mL	6.48 ± 1.55	6.36 ± 1.58	0.92	0.18
Basic E2, pg/mL	37.09 ± 18.53	36.73 ± 19.65	0.22	0.83
Basic P, ng/mL	0.57 ± 1.69	0.54 ± 0.91	0.26	0.53
Basic PRL, ng/mL	18.01 ± 11.13	17.51 ± 8.01	0.61	0.54
Basic LH, mIU/mL	6.50 ± 6.46	6.62 ± 9.18	−0.17	0.87
Basic TS, ng/mL	0.32 ± 0.21	0.35 ± 0.24	−1.61	0.054
AMH, ng/mL	4.50 ± 3.82	4.13 ± 3.33	1.18	0.24
Endometrial thickness at transfer, mm	9.01 ± 1.60	9.03 ± 1.66	−0.15	0.88

PGT-A, preimplantation genetic testing for aneuploidy; BMI, body mass index; FSH, follicle-stimulating hormone; E2, estradiol; P, progesterone; PRL, prolactin; LH, luteinizing hormone; TS, testosterone; AMH, anti-Müllerian hormone.

### Aneuploidy profile of Midway PGT-A and traditional PGT-A groups

A total of 863 blastocysts from 202 cycles in the Midway PGT-A group and 1,351 blastocysts from 261 cycles in the traditional PGT-A group were subjected to trophectoderm biopsy and chromosomal analysis. The mean number of biopsied blastocysts per cycle was significantly lower in the Midway PGT-A group than in the traditional PGT-A group (4.3 vs 6.2). The overall euploidy rate was comparable between the two groups (49.02 vs 48.56%, *P* = 0.83). When stratified by aneuploidy subtypes, distinct differences were observed (Table 2): the rate of numerical abnormalities was significantly higher in the Midway PGT-A group than in the traditional PGT-A group (14.48 vs 10.21%, *P* = 0.0024).

**Table 2:** Frequency and rates of aneuploidy subtypes in Midway PGT-A and Traditional PGT-A groups. Data are presented as *n* or as *n* (%). Intergroup comparisons were performed using the chi-square test. Statistical significance was set at *P <* 0.05.

Characteristics	Midway PGT-A Group	Traditional PGT-A Group	Statistics	
			*χ*²	*P*
Cycles, *n*	202	261		
Number of biopsied blastocysts	863	1,351		
Mean no. of biopsied blatocysts per cycle	4.3	6.2		
Euploid blastocyst rate	423/863 (49.02%)	656/1,351 (48.56%)	0.044	0.83
Numerical abnormality rate	125/863 (14.48%)	138/1,351 (10.21%)	9.17	0.0024
Structural abnormality rate	162/863 (18.77%)	352/1,351 (26.05%)	15.67	0.000
Complex abnormality rate	147/863 (17.03%)	198/1,351 (14.66%)	2.26	0.13
Amplification failure rate	6/863 (17.03%)	7/1,351 (0.52%)	0.28	0.59

PGT-A, preimplantation genetic testing for aneuploidy.

Conversely, the rate of structural abnormalities was significantly lower in the Midway PGT-A group (18.77 vs 26.05%, *P* < 0.001).

No significant between-group differences were detected in the rates of complex aneuploidy (17.03 vs 14.66%, *P* = 0.13) and amplification failure (0.70 vs 0.52%, *P* = 0.59).

### Clinical outcomes of Midway PGT-A and traditional PGT-A groups

No significant differences were observed between the Midway PGT-A group and the traditional PGT-A group in key clinical reproductive outcomes (Table 3), including clinical pregnancy rate (63.86 vs 61.68%, *P* = 0.63), implantation rate (59.41 vs 58.24%, *P* = 0.80), miscarriage rate (9.30 vs 11.18%, *P* = 0.60), live birth rate (38.61 vs 36.40%, *P* = 0.60), mean birth weight (3,338.59 ± 681.63 g vs 3,237.43 ± 563.28 g, *P* = 0.24), and newborn sex ratio (male/female: 39/39 vs 50/45, *P* = 0.73).

**Table 3:** Clinical outcomes of Midway PGT-A and traditional PGT-A groups. Data are presented as mean ± SD or as *n* (%). Intergroup comparisons were made using the chi-square test (categorical variables) or Student’s *t*-test (continuous variables). Statistical significance was set at *P* < 0.05.

Characteristics	Midway PGT-A group	Traditional PGT-A group	Statistics	
			*χ*^2^/*t*	*P*
Cycles, *n*	202	261		
Clinical pregnancy rate	129/202 (63.86%)	161/261 (61.68%)	0.23	0.63
Implantation rate	120/202 (59.41%)	152/261 (58.24%)	0.064	0.80
Miscarriage rate	12/129 (9.30%)	18/161 (11.18%)	0.27	0.60
Live birth rate	78/202 (38.61%)	95/261 (36.40%)	0.24	0.63
Mean birth weight, g	3,338.59 ± 681.63	3,237.43 ± 563.28	1.19	0.24
Newborn sex ratio (male/female)	39/39	50/45	0.12	0.73

PGT-A, preimplantation genetic testing for aneuploidy.

### Baseline characteristics, aneuploidy profile, and clinical outcomes of IVF and ICSI subgroups in Midway PGT-A group

In the Midway PGT-A group, 132 cycles underwent conventional IVF and 70 cycles underwent ICSI. Baseline characteristics were generally comparable between the two subgroups, with no significant differences in female/male age, BMI, basic endocrine parameters, AMH level, endometrial thickness, PGT-A indications, embryo survival rate, or euploidy rate (*P* > 0.05). Two statistically significant differences were noted (Table 4): the duration of infertility was significantly shorter in the IVF subgroup than in the ICSI subgroup (4.68 ± 2.74 years vs 5.45 ± 2.78 years, *P* = 0.03). The blastocyst formation rate was significantly higher in the IVF subgroup than in the ICSI subgroup (82.41 vs 67.50%, *P* = 0.0067). Regarding aneuploidy subtypes, no significant differences were detected between the IVF and ICSI subgroups in the rates of numerical abnormalities, structural abnormalities, complex aneuploidy, or amplification failure (*P* > 0.05).

**Table 4:** Baseline characteristics and aneuploidy profile of IVF and ICSI subgroups in Midway PGT-A group. Data are presented as mean ± SD or as *n* (%). Intergroup comparisons were performed using Student’s *t*-test, the Mann–Whitney U test, or the chi-square test. Statistical significance was set at *P* < 0.05.

Characteristics	IVF subgroup	ICSI subgroup	Statistics	
			*χ*^2^/*t*	*P*
Cycles, *n*	132	70		
Female age, years	33.22 ± 3.52	33.13 ± 3.54	0.17	0.43
Male age, years	33.65 ± 3.61	32.99 ± 3.52	1.25	0.11
Female BMI, kg/m^2^	23.66 ± 2.81	22.98 ± 2.84	1.63	0.52
Duration of infertility, years	4.68 ± 2.74	5.45 ± 2.78	−1.89	0.03
PGT-A indications				
Recurrent implantation failure	22/132 (16.67%)	17/70 (24.29%)	1.70	0.19
Recurrent miscarriage	104/132 (78.79%)	49/70 (70.00%)	1.92	0.17
Fetal malformation	6/132 (4.55%)	4/70 (5.71%)	0.13	0.72
Basic endocrine parameters				
FSH, mIU/mL	6.55 ± 1.55	6.34 ± 1.56	1.17	0.12
E2, pg/mL	37.83 ± 13.73	35.94 ± 13.82	1.18	0.12
P, ng/mL	0.48 ± 0.54	0.40 ± 0.33	1.45	0.074
PRL, ng/mL	18.72 ± 7.48	16.84 ± 17.02	1.38	0.085
LH, mIU/mL	6.51 ± 3.56	6.54 ± 3.58	−0.072	0.47
TS, ng/mL	0.32 ± 0.15	0.32 ± 0.26	−0.88	0.19
AMH, ng/mL	4.48 ± 2.60	4.57 ± 2.62	−0.30	0.38
Endometrial thickness, mm	9.06 ± 1.17	9.02 ± 1.88	0.19	0.43
Number of thawed embryos	587	356		
Survival rate	585/587 (99.66%)	356/356 (100.00%)	1.22	0.27
Complete survival rate	585/587 (99.66%)	356/356 (100.00%)	1.22	0.27
Number of day 3 embryos	108	160		
Blastocyst formation rate	89/108 (82.41%)	108/160 (67.5%)	7.36	0.0067
Number of biopsied blastocysts	559	304		
Mean no. of blastocysts biopsied	4.23	4.34		
Euploid blastocyst ratio	271/559 (48.48%)	152/304 (50.00%)	0.18	0.67
Numerical abnormality rate	81/559 (14.49%)	44/304 (14.47%)	0.00	0.99
Structural abnormality rate	106/559 (18.96%)	56/304 (18.42%)	0.038	0.85
Complex abnormality rate	93/559 (16.64%)	54/304 (17.76%)	0.0009	0.98
Amplification failure rate	3/559 (0.54%)	3/304 (0.99%)	0.77	0.38

PGT-A, preimplantation genetic testing for aneuploidy; BMI, body mass index; FSH, follicle-stimulating hormone; E2, estradiol; P, progesterone; PRL, prolactin; LH, luteinizing hormone; TS, testosterone; AMH, anti-Müllerian hormone.

### Clinical outcomes of IVF and ICSI groups in Midway PGT-A

In terms of clinical outcomes (Table 5), the IVF subgroup exhibited significantly better performance than the ICSI subgroup in two key indicators: clinical pregnancy rate (68.94 vs 54.29%, *P* = 0.039) and implantation rate (64.39 vs 50.00%, *P* = 0.047). There were no significant differences between the two subgroups in miscarriage rate (8.79 vs 10.53%, *P* = 0.76), live birth rate (43.18 vs 30.0%, *P* = 0.067), mean birth weight (3,365.09 ± 480.09 g vs 3,266.67 ± 489.42 g, *P* = 0.21), or newborn sex ratio (male/female: 31/26 vs 8/13, *P* = 0.20).

**Table 5:** Clinical outcomes of IVF and ICSI subgroups in Midway PGT-A group. Data are presented as mean ± SD or as *n* (%). Intergroup comparisons were made using the chi-square test (categorical variables) or Student’s *t*-test (continuous variables). Statistical significance was set at *P* < 0.05.

Outcomes	IVF subgroup	ICSI subgroup	Statistics	
			*χ*^2^/*t*	*P*
Cycles, *n*	132	70		
Clinical pregnancy rate	91/132 (68.94%)	38/70 (54.29%)	4.26	0.039
Implantation rate	85/132 (64.39%)	35/70 (50.0%)	3.93	0.047
Miscarriage rate	8/91 (8.79%)	4/38 (10.53%)	0.096	0.76
Live birth rate	57/132 (43.18%)	21/70 (30.0%)	3.35	0.067
Mean birth weight, g	3,365.09 ± 480.09	3,266.67 ± 489.42	0.80	0.21
Newborn sex ratio (male/female)	31/26	8/13	1.63	0.20

ICSI, intracytoplasmic sperm injection.

### Multivariate logistic regression analysis results of subgroup clinical pregnancy rate

The results of the multivariable logistic regression analysis for clinical pregnancy rate are presented in Table 6. Among all included covariates, two variables were significantly associated with clinical pregnancy rate (*P* < 0.05). Specifically, maternal age was a negative predictor of clinical pregnancy (*P* = 0.047). Fertilization method was also significantly associated with clinical pregnancy rate (*P* = 0.003), suggesting that compared with IVF (reference group), ICSI was associated with a lower probability of clinical pregnancy after adjusting for other confounding factors.

**Table 6:** Multivariate logistic regression analysis results of clinical pregnancy rate. Statistical significance was set at *P* < 0.05.

	SE	Wald	*P*-value	Exp (B)	95% CI for Exp (B)	
					Lower	Upper
Female age, years	0.066	3.946	0.047	0.877	0.770	0.998
Male age, years	0.058	0.136	0.712	1.022	0.912	1.145
Female BMI, kg/m^2^	0.053	1.222	0.269	0.944	0.851	1.046
Duration of infertility, years	0.050	0.231	0.631	1.024	0.928	1.130
Fertilization method	0.333	8.991	0.003	0.369	0.192	0.708
AMH, ng/mL	0.042	0.284	0.594	0.978	0.901	1.061
Endometrial thickness, mm	0.099	1.496	0.221	0.885	0.729	1.076

SE, standard error; BMI, body mass index; AMH, anti-Müllerian hormone.

## Discussion

This study aimed to evaluate the clinical validity of Midway PGT-A (testing residual vitrified embryos from initial IVF/ICSI cycles in patients who developed new PGT-A indications post-transfer failures) by comparing its aneuploidy profiling and reproductive outcomes with traditional PGT-A and to explore the impact of fertilization methods (IVF vs ICSI) on Midway PGT-A efficacy. The key findings demonstrated that Midway PGT-A yielded comparable euploidy rates and clinical outcomes to traditional PGT-A, and fertilization methods did not interfere with genetic diagnosis accuracy – with IVF even showing advantages in blastocyst formation and implantation rates in the Midway PGT-A cohort.

### Comparable euploidy rates but distinct aneuploidy subtype profiles between Midway PGT-A and traditional PGT-A

No significant difference was observed in the overall euploidy rate between the Midway PGT-A group (49.02%) and the traditional PGT-A group (48.56%), indicating that the additional vitrification–thawing cycle inherent to Midway PGT-A did not compromise embryonic chromosomal stability. Notably, the mean number of biopsied blastocysts per cycle was lower in the Midway PGT-A group (4.3 vs 6.2), a finding attributable to two clinical realities: first, Midway PGT-A patients had already undergone multiple embryo transfers in initial cycles, leaving fewer residual embryos for cryopreservation; second, traditional PGT-A protocols typically prioritize maximizing oocyte retrieval and embryo culture to expand the pool of blastocysts available for biopsy, whereas Midway PGT-A relies on limited prestored embryos from prior cycles.

A key distinction between the two groups lies in their aneuploidy subtype distributions: the Midway PGT-A group exhibited a significantly higher rate of numerical chromosomal abnormalities (14.48 vs 10.21%, *P* = 0.0024) but a significantly lower rate of structural abnormalities (18.77 vs 26.05%, *P* < 0.001). Numerically abnormal embryos (e.g. whole-chromosome gain/loss) are widely recognized as more detrimental to embryonic viability than structurally abnormal ones (e.g. translocation, deletion, and duplication), as numerical errors directly disrupt genomic dosage balance and are the primary cause of implantation failure and early miscarriage in assisted reproductive technology (ART) (Niu *et al.* 2020). This aligns with previous evidence showing that numerical abnormalities are the most common chromosomal defect in ART-associated pregnancy losses, whereas structural abnormalities are more prevalent in spontaneous pregnancies (Bettio *et al.* 2008). Notably, despite the distinct aneuploidy subtype profiles, the miscarriage rates between the two groups were comparable. This apparent paradox can be explained by the core role of PGT-A screening: both groups underwent rigorous aneuploidy detection, and only euploid embryos were selected for transfer. As such, the adverse effects of aneuploidy subtypes – including the more severe developmental impairment associated with numerical abnormalities – were eliminated before embryo transfer, resulting in equivalent clinical miscarriage risks between the two groups. It is important to emphasize that the detrimental effects of numerical abnormalities (relative to structural ones) are primarily manifested in early embryonic arrest or implantation failure, rather than clinical miscarriage; since PGT-A effectively excludes all aneuploid embryos regardless of subtype, these subtype-specific differences do not translate to divergent post-transfer outcomes.

The distinct aneuploidy subtype distribution is likely attributable to differences in patient baseline characteristics between the two cohorts. Midway PGT-A patients were predominantly indicated by post-transfer RMs (75.74%), whereas traditional PGT-A patients included a broader range of baseline indications (e.g. prepregnancy genetic risk factors such as advanced maternal age or parental chromosomal abnormalities). Given that numerical abnormalities are a well-documented key driver of ART-associated implantation failure and RM, this patient population difference may explain the higher numerical abnormality rate in the Midway PGT-A group. Collectively, these findings suggest that the observed aneuploidy subtype differences reflect the specific etiological characteristics of Midway PGT-A patients (i.e. a higher proportion with post-transfer miscarriage driven by numerical abnormalities) rather than any inherent effect of the Midway PGT-A protocol itself, and importantly, these subtype differences do not affect the final transfer outcome due to effective PGT-A screening.

### Midway PGT-A achieves equivalent clinical outcomes to traditional PGT-A despite additional vitrification–thawing

The most critical distinction between Midway PGT-A and traditional PGT-A is that Midway PGT-A blastocysts undergo two rounds of vitrification and thawing (first after initial culture and then post-biopsy), whereas traditional PGT-A blastocysts experience only one. This raised concerns about whether repeated cryopreservation would impair embryonic developmental potential. However, our results showed no significant differences between the two groups in core clinical outcomes: clinical pregnancy rate (63.86 vs 61.68%, *P* = 0.63), implantation rate (59.41 vs 58.24%, *P* = 0.80), miscarriage rate (9.30 vs 11.18%, *P* = 0.60), mean birth weight, or newborn sex ratio. These findings are consistent with accumulating evidence that repeated vitrification–thawing does not adversely affect reproductive outcomes (Hallamaa *et al.* 2021, Zhang *et al.* 2023). Taylor *et al.* (2014) reported that double-vitrified euploid blastocysts achieved a live birth rate of 50%, comparable to single-vitrified blastocysts; another study confirmed that embryos vitrified at both cleavage and blastocyst stages had similar outcomes to those vitrified once (Theodorou *et al.* 2022). Although a minority of studies suggested that repeated cryopreservation may increase pregnancy loss risk (Li *et al.* 2023), these discrepancies may stem from differences in vitrification protocols, embryo quality, or patient baseline characteristics. Our results, combined with the high survival rate of vitrified embryos (>95%), validate the safety of Midway PGT-A and provide critical clinical evidence for patients with residual frozen embryos who develop PGT-A indications post-transfer failures – avoiding the physical, emotional, and economic burdens of reinitiating a full IVF/ICSI cycle.

### Fertilization methods do not affect genetic diagnosis accuracy in Midway PGT-A, and IVF may favor improved reproductive outcomes

Traditional PGT-A protocols overwhelmingly prioritize ICSI over IVF, a recommendation rooted in concerns that residual sperm on the zona pellucida or granulosa cells around oocytes may contaminate biopsy samples and lead to misdiagnosis (Wilton *et al.* 2009, Harton *et al.* 2011). ESHRE data show that ICSI accounts for over 90% of PGT-A cycles (Harper *et al.* 2012, Coonen *et al.* 2020), reflecting this clinical consensus. However, our study found no significant differences in embryo survival rate, euploidy rate, or aneuploidy subtype distribution between IVF and ICSI subgroups in the Midway PGT-A cohort, indicating that IVF does not compromise the accuracy of chromosomal testing. This is supported by experimental evidence: Lynch *et al.* (2023) demonstrated that even 10 sperm cells in biopsy samples did not result in detectable DNA amplification, and Feldman *et al.* (2017) found that the proportion of paternal genetic material contamination was negligible (0.8% in IVF vs 0.7% in ICSI) and did not affect diagnosis outcomes.

Furthermore, the IVF subgroup exhibited significant clinical advantages over the ICSI subgroup: a higher blastocyst formation rate (82.41 vs 67.50%, *P* = 0.0067), a higher clinical pregnancy rate (68.94 vs 54.29%, *P* = 0.039), and a higher implantation rate (64.39 vs 50.00%, *P* = 0.047). To further validate these findings and control for potential confounding factors (e.g. maternal age, paternal age, BMI, duration of infertility, AMH, and endometrial thickness) that might influence clinical outcomes, we performed a multivariable logistic regression analysis with clinical pregnancy as the dichotomous dependent variable. The regression results confirmed that after adjusting for these confounding factors, fertilization method remained a significant predictor of clinical pregnancy rate (*B* = −0.997, SE = 0.333, *P* = 0.003, Exp (B) = 0.369, 95% CI: 0.192–0.708), indicating that ICSI was independently associated with a lower probability of clinical pregnancy compared with IVF. Notably, the regression analysis also identified maternal age as a significant negative predictor of clinical pregnancy (*B* = −0.131, SE = 0.066, *P* = 0.047, Exp (B) = 0.877, 95% CI: 0.770–0.998), while other covariates (paternal age, BMI, duration of infertility, AMH, and endometrial thickness) had no significant impact on clinical pregnancy outcomes (all *P* > 0.05). These benefits may be explained by two biological mechanisms. First, the IVF process retains the natural selection of sperm by the zona pellucida and corona radiata – a step bypassed by ICSI. Sperm that successfully penetrate these barriers tend to have superior functional and genetic integrity, including intact chromatin structure and mature hyaluronic acid receptors (Wang *et al.* 2018). Second, the ICSI subgroup had a significantly longer duration of infertility (5.45 vs 4.68 years, *P* = 0.03), a factor that may independently reduce reproductive potential. Important interpretive caveats must be noted when evaluating these favorable IVF-associated outcomes. Standardized blastocyst morphological grading was not recorded in this retrospective cohort, precluding adjustment for embryo quality, a well-established strong prognostic marker for implantation. Fertilization strategy was assigned according to baseline semen parameters; patients receiving ICSI inherently carried male factor infertility, a condition intrinsically linked to impaired sperm DNA integrity, compromised embryo development, and reduced blastocyst formation. Fertilization modality therefore acts only as a surrogate marker for distinct patient subgroups rather than an independent intervention, leaving unmeasured residual confounding even after multivariable adjustment. Additionally, the limited sample size of the ICSI subgroup may reduce statistical power to fully disentangle overlapping reproductive risk factors. Taken together, these findings challenge the long-standing clinical dogma that ICSI is mandatory for all PGT-A cycles. For patients without male factor infertility undergoing Midway PGT-A, IVF represents a viable and potentially more favorable fertilization alternative, provided that biopsy protocols mitigate contamination from zona-bound sperm or granulosa cells – for example, through sampling fully hatched trophoblast fragments.

### Limitations and future directions

This study has several limitations. First, this single-center retrospective study is subject to inherent selection bias and residual confounding. Although patients receiving conventional PGT-A may also present with risk factors such as advanced maternal age or RM and undergo testing in subsequent treatment cycles, the two cohorts differ fundamentally in treatment timing and reproductive history: Midway PGT-A is performed specifically after repeated failed embryo transfers, while conventional PGT-A is conducted before the first embryo transfer attempt in the current treatment cycle. This key distinction creates divergent cumulative embryo exposure and prior implantation failure burdens that cannot be fully balanced even with recorded baseline covariates. Multiple unmeasured confounders may skew comparative outcomes, including cumulative embryo exposure, underlying uterine receptivity, detailed prior implantation history, and patient self-selection bias. Additional unquantified confounding factors include subtle endometrial dysfunction, cumulative ovarian stimulation effects, and psychological stress associated with long-term infertility. Given these profound inherent clinical disparities between groups and incomplete retrospective clinical records, propensity score matching and sensitivity analyses could not be implemented to mitigate residual selection bias. Second, this study lacked adjustment for several well-recognized confounders. Endometrial preparation protocols (natural cycle versus hormone replacement therapy) and standardized blastocyst morphological grading were not systematically recorded or included in regression models. Most notably, standardized blastocyst morphological grading data were entirely unavailable for all study embryos. Embryo morphology is a robust, well-established prognostic biomarker strongly predictive of implantation potential and clinical pregnancy; omission of this covariate introduces unmeasured confounding and severely restricts the depth of comparative outcome interpretation across the two PGT-A groups.

Third, the Midway PGT-A cohort exhibited embryo-source heterogeneity. Biopsied blastocysts derived from either directly vitrified blastocysts or cleavage-stage embryos thawed and re-cultured to blastocysts. Differences in cryopreservation timing and *in vitro* culture processes may affect embryo viability, while subgroup stratification was not feasible due to sample constraints. Fourth, the relatively small ICSI subgroup sample size may weaken the statistical power of subgroup comparisons. Fifth, the interpretation of aneuploidy subtype differences remains speculative without solid mechanistic evidence. Finally, long-term offspring developmental and metabolic outcomes were not assessed, limiting the evaluation of the long-term safety of repeated vitrification involved in Midway PGT-A.

Future large-scale prospective studies with detailed embryo grading, standardized endometrial preparation stratification, and long-term offspring follow-up are needed to validate the efficacy and safety of Midway PGT-A and confirm the reproductive advantages of IVF in this clinical scenario.

## Conclusion

In conclusion, Midway PGT-A achieves comparable aneuploidy profiling and reproductive outcomes to traditional PGT-A, despite the additional vitrification–thawing cycle. Fertilization methods (IVF vs ICSI) do not affect the accuracy of chromosomal diagnosis in Midway PGT-A, and IVF confers superior blastocyst formation and implantation rates in this cohort. These findings support the clinical application of Midway PGT-A as a personalized and cost-effective strategy for patients who develop PGT-A indications after initial IVF/ICSI transfer failures, and these findings challenge the routine use of ICSI in PGT-A cycles without male factor indications.

## Declaration of interest

The authors declare that the research was conducted in the absence of any commercial or financial relationships that could be construed as a potential conflict of interest.

## Funding

This work was supported by the Henan Province Medical Science and Technique R&D Program (Grant No. SBGJ202102108).

## Author contribution statement

SS was responsible for study design, manuscript drafting and submission, and funding acquisition. JD and ZH contributed to manuscript drafting and statistical analysis of data. BZ was responsible for data collection.

## Ethics approval and consent to participate

This study was approved by the Ethics Committee of the First Affiliated Hospital of Zhengzhou University (Approval No.: 2025-KY-1975).
